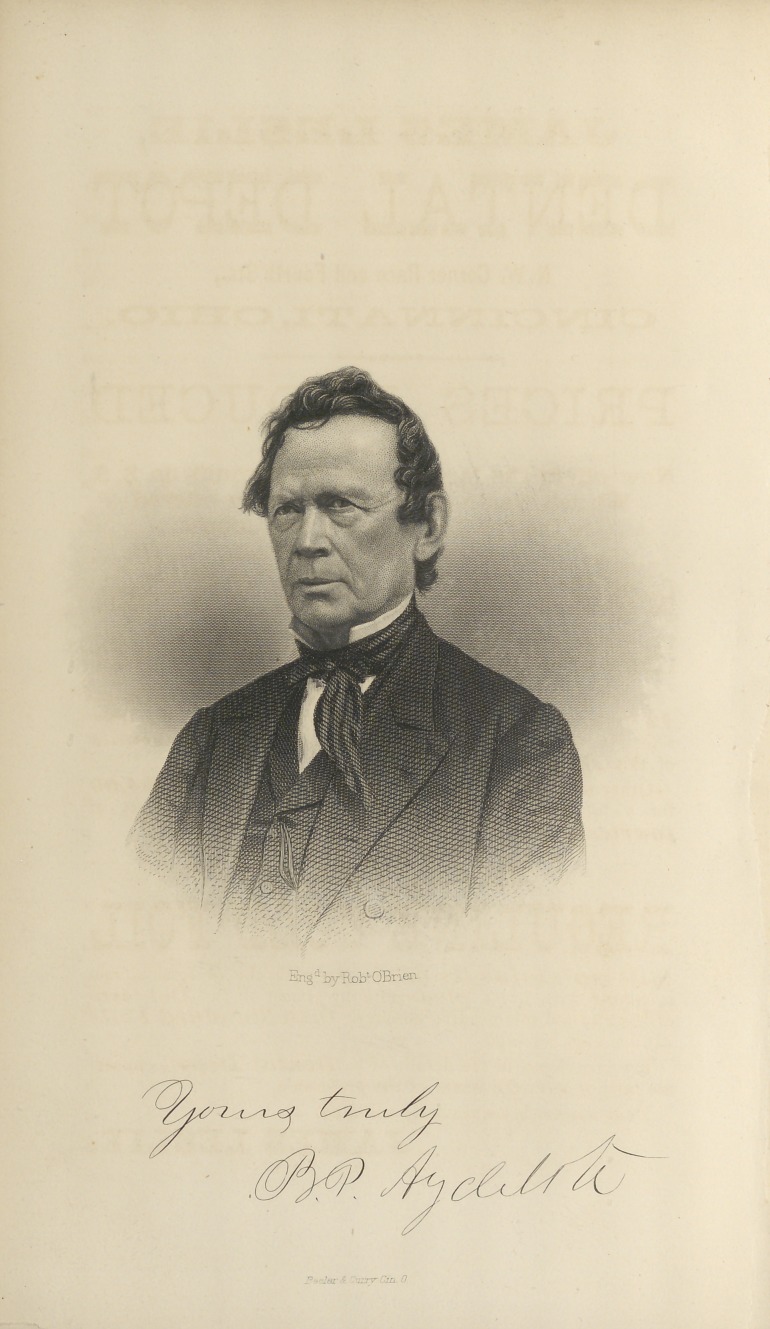# A Brief Notice of Rev. B. P. Aydelott, M. D., D. D., One of the Founders, and the First President of the Ohio College of Dental Surgery

**Published:** 1870-01

**Authors:** 


### THE DENTAL REGISTER.

VOL. XXIV.] JANUARY, 1870. [No. 1.

### Original Communications.

Surgeon Gen'l's LIBRARY 22313 Washington, D.C.

### A BRIEF NOTICE OF REV. B. P. AYDELOTT, M. D., D. D., ONE OF THE FOUNDERS AND THE FIRST PRESIDENT OF THE OHIO COLLEGE OF DENTAL SURGERY.

The gentleman whose portrait we publish in the present number of the DENTAL REGISTER was, as most of our readers know, long the President of the Board of Trustees of our College. In the charter first granted by the legislature of Ohio, in 1844, he was appointed one of the original board, and at its first meeting was unanimously elected President.

In looking back upon the history of the College we have often been constrained to regard the choice of such a head, and his long continuance in office as a remarkable evidence of the wise and benificent intentions of Providence towards the College. It would have been difficult indeed to have found another so peculiarly constituted and trained, as to give him an eminent fitness for his position.

Every intelligent observer of society must know that it is no small undertaking to found a great educational institution with nothing to begin upon, no funds, no buildings, no apparatus, and with but few competent teachers within reach. The knowledge, wisdom and patience required in those called to such a work, and the labor, the anxiety, and the sacrifices they must endure to carry it forward to a prosperous and permanent status, these must indeed be manifest to every careful, candid observer, but they can be fully known only by those who have gone through the task. Year after year did they toil on, often in darkness, and discouragement, and at times almost in despair, until at last, under the wonder-working Providence of God, a glorious success crowned their perseverance.

Such has truly been the history of our Dental College, and often have we heard Dr. Aydelott remark, when his career as the chief director of the institution has been alluded to, that "the happy result was mainly owing, under Divine blessing, to the faithful coöperation of two of the original Professors, Dr. James Taylor and Dr. Melancthon Rogers, especially to the strong common sense, the forbearance, and the practical wisdom of the former. Dr. T. has clung to the institution with a hopeful spirit, and unflagging energy in every trial, until now it has hosts of friends, and a position among the best medical schools in the land."*

We spoke of Dr. Aydelott's peculiar training as preeminently preparing him for his position in our college. In his early years he went through the full course of study in the

---

*Our readers, we doubt not, will peruse with pleasure the following very brief sketch of facts furnished by Prof. James Taylor. And equally confident are we that they will regard the want of success which Dr. Taylor and his associates met with on their application to the Faculty of the Ohio Medical College as a most happy providential disappointment. A mere professorship appended to the faculty of another college would have been of little benefit, and must have been short-lived. While thankful then to these early friends of Dental science, and the Dental profession, both they and we have ample reason to be satisfied with the result.

"After one or two interviews with some two or three members of the Faculty of the Medical College of Ohio, with reference to a chair of Practical Dentistry attached to their school, and which was thought impracticable, I first called on Dr. Rogers and opened up the subject to him, after which we called on Dr. Cook, and we then secured the charter. Drs. Rogers and Cook both going to Columbus for that purpose. These are the mere secret facts of our early organization, and I have never until now felt disposed to publish them, feeling that it was unnecessary."

medical branch of the University of the State of New York, and graduated with the strongest commendation of that able faculty. He was for upwards of ten years the President and a professor in the Woodward College, under whom it rose from the feeblest beginning to a highly prosperous condition, not only in point of numbers but of discipline and scholarship. And to say nothing of a like training in other positions, he added that varied and valuable experience which a successful pastorate of twenty-two years will be sure to give him who fills it. With these rare qualifications grafted upon studious habits, a cautious spirit, and great firmness of purpose, he came to the laborious and honorable position without the least knowledge of his appointment until he saw it in the public prints.

The result after the lapse of years of toil and solicitude now stands before the world. Our College of Dental Surgery is not merely a proud monument of the educational enterprise of Ohio, but of the enlightened confidence and cordial coöperation of the Great Valley of the Mississippi.

Many of our readers are, doubtless familiar with the annual addresses delivered by Dr. Aydelott on commencement occasions, and generally published in our journal, or in that of Baltimore. To those who have not perused them we would say, read them all faithfully, and you can not but rise up a wiser and better man—a more studious, consistent and conscientious laborer in your noble profession.

One fact will we here mention—a fact honorable alike to the College and the head thereof—he never graduates a class without placing in the hands of each member a copy of the Sacred Scriptures, as his best guide to a useful, upright, happy life here, and a glorious immortality beyond the grave. May this Christian feature ever stand out in the future history of our beloved College!

Years before Dr. Aydelott resigned his office, he frequently expressed his wish to retire, but was prevented by the urgent entreaties of the best friends of the institution. When, however, he became convinced that the profession throughout the West had arrived at such a point of improvement and elevation that it seemed best qualified to take charge of the College, he addressed the Dental Association at an Annual meeting, and recommended them to assume the responsibility of the whole work. He then retired, leaving in the most competent hands, as he believed, the important trust which he had so long endeavored faithfully to discharge.

We can add only, that Dr. Aydelott was born in the city of Philadelphia, and is now in the seventy-sixth year of his age, having survived all or nearly all his fellow students. He has seen erected every church edifice now existing in Cincinnati, and been associated as a fellow-laborer with hundreds of our city pastors, not one of whom, except himself, now remains among us. Some years since he began, one by one, to shake off every engagement and office, educational and benevolent to which the community had called him, and give his whole time and strength to the high and most responsible work of the sacred office to which he believed himself divinely called, and consecrated in his early manhood.

We are sure that all our readers will unite with us in our wishes and prayers, that his few remaining days may be crowned with increasing usefulness and happiness, and that both he and we may at last receive the gracious approval of the Righteous Judge before whom we must all shortly stand.

## Figures and Tables

**Figure f1:**